# Factors Affecting the Presence of Renal Cortical Cysts in Kidney Donors

**DOI:** 10.5152/tud.2025.24116

**Published:** 2025-01-03

**Authors:** Sevim Nuran Kuşlu Çiçek, Amil Huseynov

**Affiliations:** 1 Department of General Surgery, Biruni University, Istanbul, Türkiye; 2 Department of Transplantation, Beykoz University, Istanbul, Türkiye

**Keywords:** Transplantation, renal failure, laparoscopic surgery

## Abstract

**Objective:**

Simple renal cysts (SRCs) represent the most frequently occurring type of renal cysts, frequently observed in the elderly population. While generally considered benign, SRCs may sometimes be connected to comorbid conditions such as hypertension, aortic diseases, and renal dysfunction. This research aims to investigate the factors influencing the development of SRCs in kidney donors and the associated risks.

**Methods:**

This retrospective cohort study included 1012 living kidney donors, aged 18-87 years, who underwent renal transplant donor nephrectomy between 2008 and 2023. Data on demographic information, cyst characteristics, comorbidities, and associated risk factors were collected and analyzed using statistical methods, including Binary Logistic Regression Analysis. Renal cysts were identified and classified using computed tomography (CT) and magnetic resonance imaging (MRI) methods.

**Results:**

Renal cortical cysts were more frequently observed in males (52.76%) compared to females (47.24%), with a significant difference (*P* = .031). Donors with renal cortical cysts were significantly older (mean age 54.43 ± 12.17 years) compared to those without cysts (46.26 ± 12.35 years, *P* < .001). Substantial differences were likewise noted in fasting blood glucose, uric acid, creatinine, HbA1c, and glomerular filtration rate (GFR). The prevalence of aortic atherosclerosis was notably elevated in donors with cysts (47.74%) compared to those without (23.57%, *P *< .001). Binary logistic regression analysis indicated that older age and being male were significant factors influencing the presence of cortical cysts.

**Conclusion:**

The study confirms that SRCs are the most common renal cyst type and are more frequently observed in the elderly population. While generally benign, SRCs may be associated with increased uric acid levels and other comorbidities, suggesting potential impacts on kidney health. Additional studies are required to investigate these associations. The presence of SRCs in kidney donors is significantly associated with male gender, age, uric acid levels, and creatinine levels. These findings should be considered during the evaluation of potential kidney donors, particularly regarding the associated risks and management of SRCs.

## Introduction

Simple renal cysts (SRCs) are the most prevalent form of renal cysts and are more commonly found in the elderly population.^1^ These cysts are usually classified as Bosniak category I and II cysts.^2^ In recent years, the diagnosis of renal cysts has become easier due to advanced imaging techniques, and an increase in their prevalence has been noted.^3,4^ The literature indicates that the occurrence of SRCs rises with age and is more commonly observed in men than in women.^5,6^ In healthy populations such as kidney donors, the factors contributing to the formation of renal cysts are similar to those observed in the general population, as living donors are typically healthy volunteers. However, there is no broad consensus on the clinical significance and management of these cysts. Studies have suggested that although these cysts are generally harmless, they might sometimes be connected with comorbid conditions like high blood pressure, aortic conditions, and even renal dysfunction.^7,8^ Nonetheless, the presence of SRC has been reported not to be a barrier to kidney transplantation.^9^ This article aims to compile and discuss the potential risk factors and associated comorbidities of SRCs. Additionally, it aims to review current approaches to the monitoring and management of these cysts.

## Material and Methods

This retrospective cohort analysis was carried out utilizing the electronic medical records of Medicana International Hospital. A total of 1012 living kidney donors, aged 18-87 years, who underwent renal transplant donor nephrectomy between 2008 and 2023 were included in the research. Among these donors, 465 were male and 547 were female, with 199 donors identified as having renal cysts. Demographic information, cyst size and location, possible comorbidities, and associated risk factors of the patients were recorded. The research protocol followed the principles of the Declaration of Helsinki and received approval from the Ethics Committee of Biruni University (Approval No: 2022/76-06, Date: 02.12.2022). The demographic and laboratory data collected from all patients, along with renal computed tomography (CT) angiography examinations, were reviewed. Informed consent was obtained from the patients who agreed to take part in the study.

Renal cysts were identified using renal CT as well as magnetic resonance imaging (MRI) techniques. The cysts were classified according to the Bosniak classification (Table 1).

The medical history of patients was screened for possible comorbid conditions such as hypertension, aortic diseases, and renal dysfunction. These conditions were identified through documented diagnoses in the patients’ medical records and/or related pharmacological treatments.

The diagnosis of kidney cysts in donors was made using routine CT ANGIOGRAPHY (64-channel computed tomography scanner, VCT XTe LightSpeed General Electric, Milwaukee, Wisc., United States). An additional examination was performed with MRI in 9 patients diagnosed with Bosniak Class II.^1^ Ultrasound was not deemed necessary since all donors underwent advanced imaging as part of the standard evaluation. Additionally, the diagnosis of aortic atherosclerosis was made using renal CT angiography.

Data analysis was carried out utilizing SPSS version 25.0. (IBM SPSS Corp.; Armonk, NY, USA) Categorical data were presented as numbers and percentages and analyzed through the chi-square test. Continuous data were expressed as mean with standard deviation or median with interquartile range and assessed using either the independent t-test or Mann–Whitney U-test. A *P*-value of less than .05 was regarded as statistically significant.

The normality of variables was assessed through histogram plots and the Kolmogorov-Smirnov test. Descriptive statistics included mean, standard deviation, median, and minimum-maximum values. Categorical variables were compared via Pearson’s chi-square Test, while non-parametric variables between two groups were evaluated using the Mann–Whitney U test. The factors influencing the presence of cortical cysts were analyzed using Binary Logistic Regression. Outcomes with a *P*-value under .05 were considered statistically significant.

## Results

The study involved 1,012 kidney donors, of which 465 (45.95%) were male and 547 (54.05%) were female. The clinical and demographic data of the patients, including gender, Body Mass Index (BMI), fasting blood glucose (FBG), uric acid, creatinine, albuminuria, proteinuria, HbA1c, Glomerular Filtration Rate (GFR), age, and the presence of aortic atherosclerosis, are summarized (Table 2).

**Figure 1.**
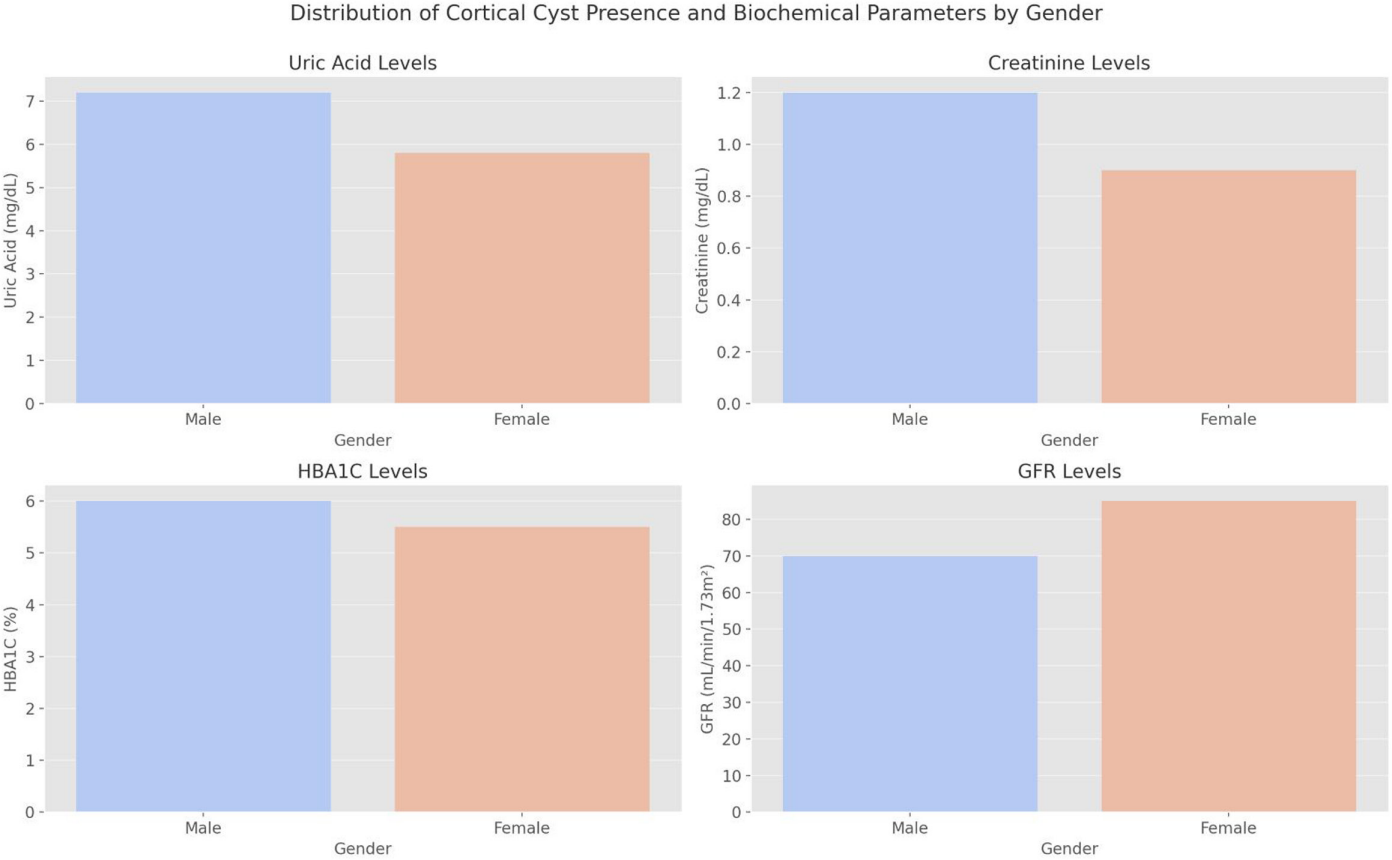
Gender-based distribution of cortical cyst presence and biochemical parameters.

The presence of renal cortical cysts was more common in males (52.76%) than females (47.24%), with a significant difference (*P* =0.031) (Figure 1). The mean age of donors with renal cortical cysts was significantly higher (54.43 ± 12.17 years) compared to those without cysts (46.26 ± 12.35 years), with a *P*-value of <0.001. No significant difference was found in BMI between donors with and without cysts (27.94 ± 4.56 vs. 27.96 ± 4.91, *P* =  0.792). However, significant differences were noted in fasting blood sugar (*P* =  0.050), uric acid (*P* =  0.016), creatinine (*P* =  0.006), HbA1c (*P* =  0.009), and GFR (*P* =  0.005). Specifically, uric acid levels were higher in those with cysts (5.09 ± 1.32 mg/dL) compared to those without (4.85 ± 1.32 mg/dL). The prevalence of aortic atherosclerosis was significantly higher in donors with cysts (47.74%) compared to those without (23.57%), with a *P*-value of <0.001.

In terms of cyst dimensions, the cysts were categorized into small (<20 mm), medium (20–50 mm), and large (>50 mm) groups. The majority of the cysts were small to medium in size, with 62% being less than 20 mm, 30% falling between 20–50 mm, and 8% being larger than 50 mm. Larger cysts (>50 mm) were more frequently associated with elevated uric acid levels (*P* =  0.014) and aortic atherosclerosis (*P* =  0.002) compared to smaller cysts.

In addition to significant differences in gender and age, it was found that uric acid levels were relatively high, and the *P*-value was indicated. Non-significant differences were observed in BMI, HbA1c, and other parameters.

### Surgical Selection for Donor Nephrectomy

In cases where donors had unilateral kidney cysts, the nephrectomy was performed on the side with the cyst. For donors with bilateral kidney cysts, standard side selection criteria were applied for the nephrectomy. The cysts were excised from the wall and transplanted. Given the low risk of malignancy, they were not subjected to specific pathological examination. No malignancy development was detected in the transplanted kidneys with cysts.

### Comparative Analysis Based on Cortical Cyst Presence

The comparative analysis revealed that individuals with cortical cysts had an elevated proportion of males, greater uric acid and creatinine levels, while HbA1c and GFR levels were lower. Additionally, individuals with cortical cysts were older and exhibited a higher prevalence of aortic atherosclerosis.

### Binary Logistic Regression Analysis

The binary logistic regression analysis of the patients’ clinical and demographic information is shown in Table 3. Univariate analysis results indicated that gender, uric acid, GFR, age, and presence of aortic atherosclerosis were statistically significant, whereas in multivariate analysis, only age and gender remained significant. According to the results of our study, each year of age increases the presence of cortical cysts by 1.046 times, and male gender increases the presence of cortical cysts by 1.314 times.

## Discussion

This study provides a comprehensive investigation into the occurrence rate, contributing factors, and associated comorbidities of SRC. The literature indicates that the occurrence of SRC rises with age and is generally clinically insignificant.^3^ However, some existing studies have shown that basic renal cysts can be linked to comorbidities like hypertension and aortic diseases.^7,8^ Examining these potential links provides a broader understanding of the possible impacts of SRCs.

Additionally, there is evidence suggesting that SRC may increase the risk of renal dysfunction.^10^ This necessitates a further evaluation of the impact of SRC on patients. In our research, we explored the connection between the presence of SRCs and serum uric acid levels. Our results indicate that patients with renal cysts have markedly elevated serum uric acid levels in comparison to the control group. This finding aligns with findings from other studies in the literature, supporting the potential impact of SRCs on uric acid metabolism.^11-14^

Nevertheless, there is no consensus on the clinical significance of SRC. Some studies have reported that the presence of SRC is not an obstacle for kidney donor selection for transplantation.^9^ This suggests that basic renal cysts are generally harmless and they do not typically necessitate intervention.^11^

Our findings align with the literature, confirming that basic renal cysts represent the most common form of renal cysts and are more commonly observed among older adults.^12^ Furthermore, our study provides evidence that basic renal cysts might be linked to renal impairment, although this relationship remains controversial. Particularly, studies by Simms RS and colleagues have indicated that the presence of SRC in kidney donors does not preclude them from being kidney donors.^13^ The connection between the presence of SRCs and uric acid levels offers a fresh outlook on the medical significance of these cysts. The increase in uric acid levels may indicate potential effects of renal cysts on kidney health, which should be further explored in more extensive studies.

In this study, we observed a higher prevalence of renal cysts in male donors compared to female donors. This finding is consistent with other studies that suggest that hormonal differences, particularly the influence of androgens, may contribute to this gender disparity. Additionally, genetic predisposition and lifestyle factors commonly associated with males, such as higher rates of hypertension and metabolic disorders, may also play a role in the increased occurrence of renal cysts. Further research is necessary to explore these potential mechanisms and confirm the underlying reasons for this gender difference.

It is well-established that elevated uric acid levels are associated with an increased risk of kidney stone formation. However, in our research, despite the observed higher uric acid levels in patients with renal cysts, we did not detect any pathological stone formation. This may be due to the relatively short follow-up period or the specific characteristics of our study population, which consisted of otherwise healthy kidney donors. Further longitudinal studies would be beneficial to determine whether there is a delayed onset of stone formation in individuals with elevated uric acid and renal cysts.

The findings of our research indicate that the presence of SRC is significantly associated with male gender, age, uric acid, and creatinine levels. In contrast, HbA1c and GFR values were found to have less impact on cyst formation. A consensus has yet to be reached on the clinical significance regarding simple kidney cysts. While these cysts are generally considered benign, they may be associated with comorbidities or increase the risk of renal dysfunction in certain situations.

On the other hand, this study confirms that basic renal cysts are commonly found in living kidney donors and are significantly associated with factors such as gender, age, uric acid levels, GFR, and the presence of aortic atherosclerosis. These findings should be considered in the evaluation process of potential kidney donors.

## Data Availability Statement:

The data of this study is available upon request to the corresponding author.

## Figures and Tables

**Table 1.** The Diagnosis of Renal Cysts in Donors Was Made by Routine Computed Tomography Angiography (64-Channel Computed Tomography Scanner [VCT XTe LightSpeed, General Electric, Milwaukee, Wisc., United States]). Additional Examination Was Performed with MRI in 9 Patients with Bosniak Classic II (1).

CT	MRI
Six types, all homogeneous and well-defined with thin (≤2 mm) smooth walls: 1. Cystic masses with thin (≤2 mm) and few (1-3) enhancing septa; any non-enhancing septa; may have calcifications of any type. 2. Noncontrast CT: −9 to 20 HU 3. Noncontrast CT: ≥70 HU 4. Contrast CT: non-enhancing masses >20 HU at renal mass protocol CT, may have calcification of any type. 5. Contrast CT: masses 21-30 HU at portal venous phase CT 6. Homogeneous low-attenuation masses that are too small to characterize	1. Like point 1 of CT-criteria or 2. Homogeneous masses markedly hyperintense at T2WI similar to CSF at non-contrast MRI. 3. Homogeneous masses markedly hyperintense at T1WI at non-contrast MRI (approx 2.5 normal parenchymal signal intensity).

**Table 2.** Clinical and Demographic Data in Relation to Cortical Cyst Presence

Description	Total (N = 1012)	Absent (N = 813)	Present (N = 199)	*P*¹
Gender				.031²
Male	465 (45.95%)	360 (44.28%)	105 (52.76%)	
Female	547 (54.05%)	453 (55.72%)	94 (47.24%)	
BMI (kg/m²)	27.96 ± 4.84	27.96 ± 4.91	27.94 ± 4.56	.792
Fasting Blood Sugar (mg/dL)	94.84 ± 10.83	94.66 ± 11.21	95.53 ± 9.21	.050
Uric Acid (mg/dL)	4.9 ± 1.32	4.85 ± 1.32	5.09 ± 1.32	.016
Creatinine (mg/dL)	0.75 ± 0.19	0.75 ± 0.20	0.77 ± 0.11	.006
Albuminuria (mg/L)	12.21 ± 10.70	12.36 ± 11.05	11.65 ± 9.32	.665
Proteinuria (mg/L)	94.28 ± 49.55	92.31 ± 48.10	101.39 ± 54.02	.109
HBA1C (%)	5.78 ± 4.21	5.8 ± 4.71	5.72 ± 0.50	.009
GFR (mL/min/1.73m²)	120.9 ± 31.01	122.05 ± 31.79	116.16 ± 27.10	.005
Age (years)	47.87 ± 12.73	46.26 ± 12.35	54.43 ± 12.17	<.001
Aortic Atherosclerosis				<.001²
Absent	717 (71.63%)	613 (76.43%)	104 (52.26%)	
Present	284 (28.37%)	189 (23.57%)	95 (47.74%)	

^1^Mann–Whitney U-test; ^2^Chi-square test.

This table clearly presents the comparison of clinical and demographic data between patients with and without cortical cysts, including gender, BMI, fasting blood sugar, uric acid, creatinine, albuminuria, proteinuria, HBA1C, GFR, age, and the presence of aortic atherosclerosis. Statistical significance is indicated by *P*-values.

**Table 3.** Analysis of Factors Affecting the Presence of Cortical Cysts

Variable	Univariate Analysis	Multivariate Analysis
	B	Sig.
Gender (Male)	0.340	0.032
Uric Acid	0.138	0.022
Creatinine	0.460	0.234
HbA1C	−0.006	0.811
GFR	−0.007	0.017
Age	0.052	<0.001
Aortic atherosclerosis (present)	1.086	<0.001

Binary Logistic Regression Analysis

This table presents the results of a binary logistic regression analysis, examining the influence of various factors on the presence of cortical cysts. It includes both univariate and multivariate analysis results, showing the regression coefficient (B), significance (Sig.), and the odds ratio (Exp(B)) with its confidence interval (Lower-Upper). The factors analyzed include gender, uric acid, creatinine, HbA1C, GFR, age, and the presence of aortic atherosclerosis.
